# Heart Rate Variability Predicts Therapeutic Response to Metoprolol in Children With Postural Tachycardia Syndrome

**DOI:** 10.3389/fnins.2019.01214

**Published:** 2019-11-12

**Authors:** Yuanyuan Wang, Chunyu Zhang, Selena Chen, Ping Liu, Yuli Wang, Chaoshu Tang, Hongfang Jin, Junbao Du

**Affiliations:** ^1^Department of Pediatrics, Peking University First Hospital, Beijing, China; ^2^Research Unit of Clinical Diagnosis and Treatment of Pediatric Syncope and Cardiovascular Diseases, Chinese Academy of Medical Sciences, Beijing, China; ^3^Division of Biological Sciences, University of California, San Diego, San Diego, CA, United States; ^4^Key Laboratory of Molecular Cardiovascular Sciences, Ministry of Education, Beijing, China; ^5^Department of Physiology and Pathophysiology, Health Sciences Centre, Peking University, Beijing, China

**Keywords:** heart rate variability, children, postural tachycardia syndrome, metoprolol, therapy

## Abstract

**Purpose:**

To improve the metoprolol therapeutic effectiveness, we aimed to explore whether baseline heart rate variability (HRV) indicators before metoprolol treatment were useful for predicting its efficacy for postural tachycardia syndrome (POTS).

**Methods:**

We recruited 45 children with POTS who received metoprolol and 17 healthy controls. All children underwent a standing test or basic head-up tilt test and 24-h dynamic electrocardiography before treatment. After 3 months of metoprolol, therapeutic responsiveness was evaluated. The usefulness of baseline HRV parameters in predicting the effectiveness of metoprolol was studied and the long-term cumulative symptom rate was analyzed.

**Results:**

The baseline HRV frequency domain indicators for power, ultra-low frequency, very-low frequency, low frequency (LF), high frequency (HF), and total power (TP) as well as time domain indicators were significantly lower for responders than non-responders to metoprolol; however, low-frequency normalized units and LF/HF ratio were markedly greater for responders than non-responders. On series-parallel analysis, combined baseline triangular (TR) index ≤ 33.7 and standard deviation of all normal-to-normal intervals (SDNN) index ≤ 79.0 ms as cut-off values yielded sensitivity, specificity and accuracy of 85.3, 81.8, and 84.4%, respectively, to predict therapeutic responsiveness to metoprolol. On long-term follow-up, the cumulative symptom rate was significantly higher with TR index > 33.7 and SDNN index ≤ 79.0 ms, TR index ≤ 33.7 and SDNN index > 79.0 ms or TR index > 33.7 and SDNN index > 79.0 ms than TR index ≤ 33.7 and SDNN index ≤ 79.0 ms (*P* < 0.05).

**Conclusion:**

Combined TR index ≤ 33.7 and SDNN index ≤ 79.0 ms were useful preliminary measures to predict therapeutic response to metoprolol in pediatric POTS.

## Introduction

Postural tachycardia syndrome (POTS) is a common type of orthostatic intolerance (OI) and is characterized by excessive increase in heart rate (HR) when standing upright (Fedorowski et al., 2017). POTS is accompanied by symptoms of OI (Fedorowski, 2019) and results in serious physical and psychological problems in children (Bagai et al., 2011; Kizilbash et al., 2014). A beta-adrenoceptor blocker (β-blocker) is commonly used for treating POTS in children (Kernan and Tobias, 2010; Bryarly et al., 2019). It inhibits sympathetic nerve modulation, reducing HR and the stimulation of cardiac baroreceptors, thus blocking the action of increased catecholamine levels in circulation. Previous studies showed that β-blockers could improve symptoms only in some children with POTS (Lai et al., 2009; Chen et al., 2011). Additionally, β-blockers may impair the exercise tolerance of children (Ladage et al., 2013). Therefore, predicting the therapeutic effect of the β-blocker metoprolol on POTS before treatment is of great clinical importance to improve the effectiveness of the therapy.

In previous studies, we examined predictive indices of metoprolol efficacy before treating patients with POTS by detecting plasma levels of norepinephrine, copeptin and C-type natriuretic peptide (CNP) (Zhang et al., 2014; Zhao et al., 2014; Lin et al., 2015b). However, norepinephrine level in plasma is unstable, which limits its predictive accuracy, and the detection of plasma norepinephrine, copeptin, and CNP requires venipuncture to obtain blood samples for ELISA. Therefore, non-invasive and easy-to-measure indices are needed for predicting the therapeutic efficacy of metoprolol before POTS treatment.

HR variability (HRV) is an important measure that reflects the sympathetic and vagal modulation of the autonomic nervous system and its balance (Cygankiewicz and Zareba, 2013). It is primarily measured by 24-h dynamic Holter electrocardiography, which is non-invasive and easy-to-operate. Therefore, to improve the therapeutic effectiveness of metoprolol for pediatric POTS, we aimed to determine useful baseline HRV-based parameters to predict its efficacy for POTS.

## Materials and Methods

### Study Population

From March 2012 to August 2018, 45 children with POTS admitted to the Pediatric Syncope Clinic of Peking University First Hospital were enrolled in the POTS group (23 males; mean age 12.2 ± 2.2 years). All received metoprolol. The control group consisted of 17 healthy children (11 males, mean age 11.5 ± 2.0 years) screened by medical history, physical examination, and laboratory investigations including ECG, Holter ECG, standing test, etc. This study was approved by the Ethics Committee of the First Hospital of Peking University (2018 [202]), and all parents or guardians of the children were informed of the research purpose and signed informed consent.

### HRV Indices Analysis (Malik et al., 1989; Myers et al., 1992; Nguyen et al., 2017)

Heart rate variability was assessed by 3-channel 24-h Holter ECG (Mortara Dynamic ECG Recording Analyzer, United States), with sampling rate 10000 Hz and response band 0.05 to 60 Hz. All participants were required to be hospitalized during the recording and away from electronic products. Sitting, reading, walking, eating snacks, and drinking tea was allowed at the bedside. HRV indices were analyzed by using H scribe (Mortara Instruments). Each RR interval was validated visually before the analysis. Only normal-to-normal (NN) beats were considered for analysis with intervals. Interfering signal exclusion was performed by the analysis system automatically based on the normalized QRS peak detection, and abnormal heart beats were screened by an investigator who was blinded to the results. Abnormal heart beats included ventricular or supraventricular heart beats, artifacts and noise. We also excluded the recordings that provided < 20 h of usable data (≥240 of 288 5-min segments), requiring for time-domain analyses that at least 50% of each segment consisted of NN inter-beat intervals and for frequency-domain analyses at least 80%. After we checked manually, an automatic algorithm was applied to select the most stationary segments of 5-min duration, and 403 ± 92 beats were selected in each series. The time domain parameters were as follows: standard deviation of all NN intervals (SDNN), standard deviation of the averages of NN intervals in all 5-min segments of the entire recording (SDANN), mean of the standard deviation of NN intervals for each 5-min segment (SDNN index), root mean square of the successive NN interval difference (RMSSD), percentage difference between adjacent NN intervals > 50 ms (pNN50) and triangular (TR) index.

The frequency domain parameters of HRV were calculated by spectral analysis performed by Fast Fourier Transform methods. Recordings were detrended and low-pass–filtered to remove frequencies > 60 Hz. The power of frequency bands could be classified into four bands: ultra-low frequency (ULF; 0–0.003 Hz), very low frequency (VLF; 0.003–0.04 Hz), low frequency (LF; 0.04–0.15 Hz), high frequency (HF; 0.15–0.40 Hz), total power (TP; variance of all NN intervals, ≤0.4 Hz), and ratio of LF to HF (LF/HF). We defined 22:00–05:59 as the night time, and nighttime HRV indices were calculated. Participants were also asked to refrain from strenuous exercise and emotional excitement.

### Standing Test and Basic Head-Up Tilt Test (BHUTT)

#### Standing Test (Liao et al., 2010; Wang et al., 2018)

The test environment requires dim light and a suitable temperature. The children first laid quietly for 10 min. The HR, blood pressure (BP), and ECG recordings were monitored by using a Dash 2000 multi-channel physiological monitor (General Electric, Co., New York, NY, United States) while children were lying supine. After HR and BP stabilized, children were then asked to stand for another 10 min, and HR, BP and ECG recordings were monitored dynamically during this process.

#### BHUTT (Lin et al., 2015a; Wang et al., 2018)

All drugs affecting autonomic function were discontinued for least five half-lives before the test. Children were required to fast for > 4 h before the test. Children were first asked to assume a supine position on the tilt bed (HUT-821; Beijing Juchi, Beijing) for 10 to 30 min. HR and ECG recordings were continuously monitored in a quiet, warm, and dim light environment with a multi-lead ECG monitor (General Electric, New York, NY, United States), and BP was monitored by using Finapres Medical System- FMS (FinometerPRO, FMS Company, Netherlands). After HR and BP stabilized, the tilt bed was raised to 60°, and HR, BP and ECG recordings were monitored until a positive reaction or until the children completed the entire 45-min examination.

### Diagnosis of POTS

The diagnosis of POTS was mainly based on the following (Sheldon et al., 2015; Wang et al., 2018, 2019): (1) commonly seen in older children; (2) associated with inducements such as quick position change from supine to upright position, or long-term standing before the appearance of OI symptoms; (3) associated with OI symptoms such as dizziness, headache, fatigue, blurred vision, chest tightness, palpitations, hand tremors, and even syncope; (4) HR increased ≥ 40 bpm or the maximum HR ≥ 130 bpm (in children 6–12 years old) or ≥ 125 bpm (in adolescents 13–18 years old) without orthostatic hypotension (BP decrease > 20/10 mmHg) during the first 10 min of the standing test or BHUTT; and (5) exclusion of other diseases that cause OI symptoms such as cardiovascular diseases, metabolic diseases, neurologic diseases, or psychogenic disorders.

### Symptom Score (SS) (Winker et al., 2005; Li et al., 2016)

SS was based primarily on symptoms of OI in children with POTS, including syncope, dizziness, chest tightness, nausea, palpitations, headache, hand tremors, sweating, blurred vision, and inattention. Scoring criteria were based on the frequency of an event during the observation: (0) no occurrence; (1) once per month; (2) 2 to 4 times per month; (3) 2 to 7 times per week; and (4) more than once per day. A child’s total SS was the sum of his/her individual SS. The baseline SS was determined when the child was first diagnosed with POTS before treatment, and it was recorded again at the end of the first follow-up after treatment. A reduction in score by ≥ 2 points after the treatment as compared to baseline SS indicated that the treatment was effective and the patient was considered a “responder.” Otherwise, an SS reduction < 2 points indicated that the treatment was ineffective, and the patient was considered a “non-responder.”

### Treatment and Follow-Up

#### First Follow-Up

Children with POTS received metoprolol. The standard dosage was 12.5 mg twice a day, but for a few children the dose was adjusted according to age and weight. The course of treatment was 1 to 3 months. After 3 months of treatment, children were followed up for the first time. The follow-up was conducted by questionnaire implemented in an outpatient setting or via telephone and was recorded by a trained responsible person. Drug adherence, frequency of OI symptoms and adverse drug reactions were the focus of the follow-up.

#### Second Follow-Up

**FIGURE 1:**
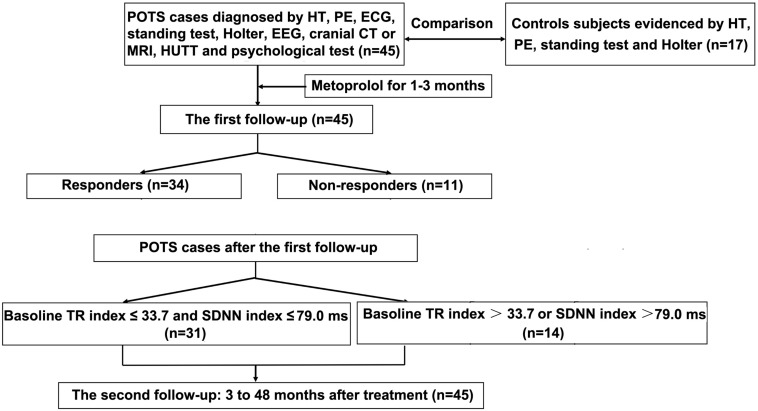
Flowchart of enrollment of study population. POTS, postural tachycardia syndrome; HT, history taking; PE, physical examination; ECG, electrocardiography; EEG, electroencephalography; CT, computed tomography; MRI, magnetic resonance imaging; HUTT, head-up tilt test; TR index, triangular index.

The second telephone follow-up time was scheduled from January to February 2019. The follow-up involved Kaplan–Meier curve analysis of children with POTS at 3 to 48 months after discontinuation of treatment. The flowchart of study enrollment is in Figure 1. According to the cut-off values for predicting the therapeutic response to metoprolol in the first follow-up, together with the symptom scores before and after treatment, we plotted the Kaplan–Meier curve to determine whether the cut-off values derived from the first follow-up could predict the long-term therapeutic response to metoprolol in children with POTS.

### Statistical Analysis

SPSS 23.0 (IBM, Armonk, NY, United States) was used for data analysis. Continuous data are expressed as mean ± SD. The data normality test was performed using the Shapiro–Wilk test. Independent sample *t*-test or Mann–Whitney *U*-test was used to compare other data between the two groups, and chi-square test was used for comparing categorical data. The Pearson correlation test was used to examine the correlation between normal distribution indices and the Spearman correlation test was used to examine the correlation between non-normal distribution indices. Receiver operator characteristic (ROC) curve analysis was used to evaluate the sensitivity and specificity of HRV indicators for predicting the short-term efficacy of metoprolol in children with POTS. An area under the ROC curve (AUC) 0.5 to 0.7 represented “low” predictive power, 0.7 to 0.9 “moderate” predictive power, and > 0.9 “high” predictive power. The optimal cutoff value was determined by the maximum Youden index, defined as sensitivity + specificity – 1, with sensitivity and specificity calculated as proportions (Schisterman et al., 2005).

According to the first follow-up-derived indices, children with POTS were divided into two groups. The OI symptoms and follow-up times were recorded at the second follow-up. The Kaplan–Meier curve was drawn, and symptom survival rate was compared by Log-rank test. The symptom rate over a certain period of time was defined as P (t) = 1 – (the number of asymptomatic cases after treatment that used to be symptomatic during this time/the number of cases observed at the beginning of this follow-up) × 100%, and cumulative symptom rate was calculated as S(t) = P(1) × P(2) × … × P (t). Statistical significance was set at *P* < 0.05.

## Results

### Comparison of Baseline Demographic and HRV Parameters Between POTS and Control Groups

The two groups did not significantly differ in sex, age, height, weight and body mass index (BMI) (*P* > 0.05) (Table 1). However, SDNN index, pNN50, TR index, LF, and TP were significantly higher in POTS than control children all *P* < 0.05. The other HRV indicators were not significantly different between the two groups (*P* > 0.05) (Table 2).

**TABLE 1:** Demographic characteristics of children with postural tachycardia syndrome (POTS) and control groups.

**Groups**	**Case (*n*)**	**Sex (M/F)**	**Age (y)**	**Height (cm)**	**Weight (kg)**	**BMI (kg/m^2^)**
POTS	45	23/22	12.2 ± 2.2	158.0 ± 13.7	50.2 ± 17.1^a^	19.6 ± 4.2^a^
Control	17	11/6	11.5 ± 2.0	155.7 ± 11.9	48.3 ± 11.7	19.7 ± 2.9
t/χ^2^/Z	–	0.454	−1.235	−0.614	−0.197	−1.271
*P*	–	0.501	0.221	0.541	0.848	0.207

*Data are mean ± SD. ^*a*^Non-normal distribution.*

**TABLE 2:** Comparison of HRV indices between POTS and control groups.

**Groups**	**SDNN (ms)**	**SDANN (ms)**	**SDNN index (ms)**	**RMSSD (ms)**	**pNN50 (%)**	**TR index**	**ULF (ms^2^)**	**VLF (ms^2^)**	**LF (ms^2^)**	**HF (ms^2^)**	**TP (ms^2^)**	**LF/HF**
POTS	143.2 ± 33.9	133.7 ± 42.5^a^	68.4 ± 16.9	49.3 ± 20.5^a^	20.6 ± 11.4	29.4 ± 7.3	15650.7 ± 7954.0^a^	2443.7 ± 965.3^a^	1075.3 ± 561.7^a^	901.4 ± 756.6^a^	3384.9 ± 1774.1^a^	1.6 ± 0.8^a^
Control	130.6 ± 31.7	144.5 ± 72.9^a^	55.0 ± 13.5	38.4 ± 13.2	14.4 ± 8.6	24.5 ± 7.0	13801.6 ± 8333.4^a^	1954.3 ± 856.2	747.7 ± 401.3^a^	554.7 ± 388.5^a^	2395.2 ± 1175.4^a^	1.6 ± 0.7^a^
t/Z	−1.317	−0.253	−2.934	−1.879	−2.038	−2.356	−1.034	−1.633	−2.012	−1.649	−1.996	−0.395
*P*	0.193	0.806	0.005	0.060	0.046	0.022	0.308	0.105	0.044	0.101	0.046	0.699

*Data are mean ± SD. HRV, Heart rate variability; SDNN, standard deviation of all NN intervals during 24-h recording; SDANN, standard deviation of the average of NN intervals in all 5-min segments of the entire recording; SDNN index, mean of the standard deviation of NN intervals for each 5-min segment; RMSSD, root mean square of the successive NN interval difference; pNN50, percentage of adjacent RR intervals > 50 ms; TR index, triangular index; ULF, ultra low frequency; VLF, very low frequency; LF, low frequency; HF, high frequency; TP, total power; LF/HF, ratio of low to high frequency power. Data are mean ± SD. ^*a*^Non-normal distribution.*

### Comparison of Baseline Demographic, Hemodynamics, Pre-treatment SS and HRV Parameters Between Responders and Non-responders to Metoprolol in Children With POTS

The two response groups did not significantly differ in sex, age, height, weight, BMI, systolic BP, diastolic BP, HR, and HR change from supine to standing (ΔHR), or pre-treatment SS (*P* > 0.05) (Table 3). The time domain indices, ULF, VLF, LF, HF, and TP, were markedly lower in responders than in non-responders; however, LF/HF were significantly higher in responders than non-responders (*P* < 0.05 for SDANN and LF/HF, and *P* < 0.01 for other parameters) (Table 4). Night-time HRV indices did not significantly differ between responders and non-responders (Table 5).

**TABLE 3:** Comparison of demographic, hemodynamics parameters and pre-treatment SS (symptom scores) between POTS children with response and non-response to metoprolol.

**Groups**	**Cases (*n;* %)**	**Sex (M/F)**	**Age (y)**	**Height (cm)**	**Weight (kg)**	**BMI (kg/m^2^)**	**HR (bpm)**	**SBP (mmHg)**	**DBP (mmHg)**	**ΔHR (bpm)**	**Pre-treatment SS (point)**
Responders	34 (75.6)	18/16	12.0 ± 2.3	156.8 ± 15.2	49.8 ± 18.3	19.6 ± 4.3^a^	78 ± 13^a^	109 ± 12	63 ± 11	48 ± 8	6.6 ± 3.9^a^
Non-responders	11 (24.4)	5/6	12.9 ± 1.6	161.6 ± 7.2	51.4 ± 13.4	19.5 ± 4.1	74 ± 15	105 ± 8	62 ± 9	47 ± 8^a^	7.1 ± 4.4
t/Z/χ2	–	0.007	1.201	1.015	0.259	−0.092	−1.375	−0.805	−0.183	−0.595	−0.346
*P*-value	–	0.932	0.236	0.316	0.797	0.926	0.169	0.425	0.855	0.552	0.729

*HR, heart rate; SBP, systolic blood pressure; DBP, diastolic blood pressure; ΔHR, heart rate change from supine to standing. Data are mean ± SD. ^*a*^Non-normal distribution.*

**TABLE 4:** Comparison of HRV indices between POTS children with response and non-response to metoprolol.

**Groups**	**SDNN (ms)**	**SDANN (ms)**	**SDNN index (ms)**	**RMSSD (ms)**	**pNN50 (%)**	**TR index**	**ULF (ms^2^)**	**VLF (ms^2^)**	**LF (ms^2^)**	**HF (ms^2^)**	**TP (ms^2^)**	**LF/HF**
Responders	134.6 ± 32.6^a^	128.4 ± 44.4^a^	63.2 ± 12.8	43.2 ± 15.2	17.5 ± 9.6	27.3 ± 6.1	14083.5 ± 8129.1^a^	2196.2 ± 770.7	883.4 ± 340.6	665.4 ± 478.2^a^	2811.5 ± 1147.9^a^	1.7 ± 0.8^a^
Non-responders	169.5 ± 23.5^a^	150.3 ± 32.3^a^	84.5 ± 18.3	67.9 ± 24.2	30.2 ± 11.5	35.7 ± 7.2	20494.8 ± 5112.6	3208.8 ± 1134.9	1630.7 ± 994.8	1630.7 ± 994.8	5257.3 ± 2219.8	1.2 ± 0.5
t/Z	−3.078	−2.206	4.305	4.013	3.634	3.779	−2.958	−2.588	5.013	−3.222	−3.143	−2.166
*P*	0.002	0.027	<0.001	<0.001	0.001	<0.001	0.003	0.010	<0.001	0.001	0.002	0.030

*HRV, Heart rate variability; SDNN, standard deviation of all NN intervals during 24-h recording; SDANN, standard deviation of the average of NN intervals in all 5-min segments of the entire recording; SDNN index, mean of the standard deviation of NN intervals for each 5-min segment; RMSSD, root mean square of the successive NN interval difference; pNN50, percentage of adjacent RR intervals > 50 ms; TR index, triangular index; ULF, ultra low frequency; VLF, very low frequency; LF, low frequency; HF, high frequency; TP, total power; LF/HF, ratio of low to high frequency power. Data are mean ± SD. ^*a*^Non-normal distribution.*

**TABLE 5:** Comparison of HRV indices for the night between POTS children with response and non-response to metoprolol.

**Groups**	**nSDNN (ms)**	**nRMSSD (ms)**	**nPNN50 (%)**	**nTR index**	**nULF (ms^2^)**	**nVLF (ms^2^)**	**nLF (ms^2^)**	**nHF (ms^2^)**	**nTP (ms^2^)**	**nLF/HF**
Responders	73.1 ± 19.9	58.1 ± 25.3^a^	33.0 ± 17.0	11.7 ± 2.5	24.4 ± 7.1	999.0 ± 290.3	712.7 ± 280.5	909.5 ± 696.5^a^	2308.7 ± 1029.5	1.3 ± 0.6^a^
Non-responders	88.3 ± 30.1	80.3 ± 40.8	44.3 ± 20.8	13.3 ± 4.2	22.8 ± 6.0	997.4 ± 304.8	893.8 ± 496.9	1594.7 ± 1273.5	3188.5 ± 1920.3	1.2 ± 1.1^a^
t/Z	1.564	−1.664	1.812	1.270	−0.686	−0.016	1.522	−1.611	1.453	−1.604
*P*	0.142	0.096	0.077	0.228	0.497	0.988	0.135	0.107	0.172	0.109

*HRV, Heart rate variability; nSDNN, night standard deviation of all NN intervals during 24-h recording; nRMSSD, night root mean square of the successive NN interval difference; npNN50, night percentage of adjacent RR intervals > 50 ms; nTR index, night triangular index; nULF, night ultra low frequency; nVLF, night very low frequency; nLF, night low frequency; nHF, night high frequency; nTP, night total power; nLF/HF, night ratio of low to high frequency power. Data are mean ± SD. ^*a*^Non-normal distribution.*

### Baseline HRV Time-Domain Indices Predict Short-Term Therapeutic Response to Oral Metoprolol in Children With POTS

**FIGURE 2:**
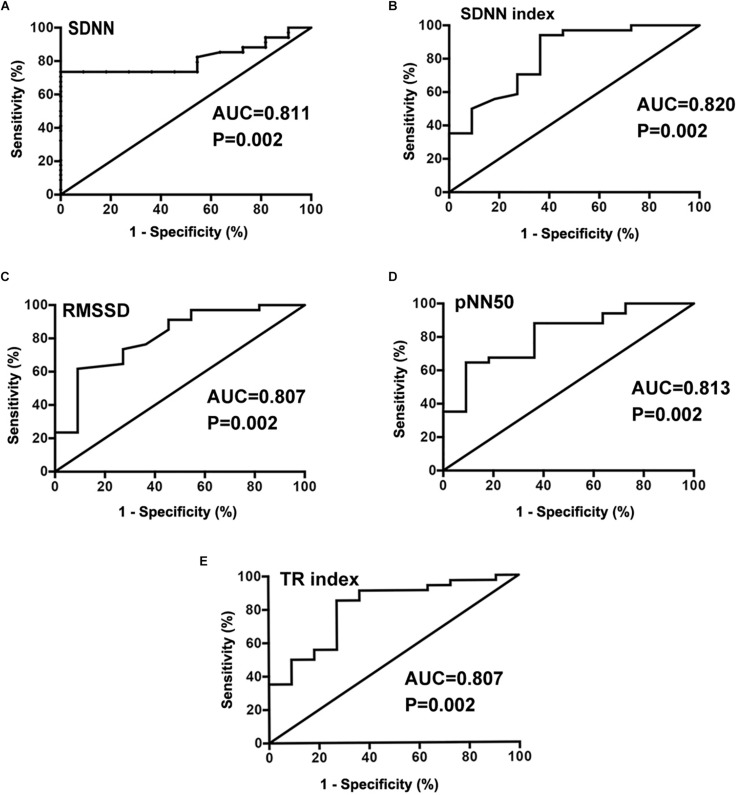
Receiver operating characteristic (ROC) curve analysis of HRV time domain indexes of SDNN_(log__2__)_ **(A)**, SDNN index **(B)**, RMSSD **(C)**, pNN50 **(D)**, and TR index **(E)** as predictors of therapeutic response to metoprolol in children with POTS.

Before ROC curve analysis, the non-normal distribution of SDNN, SDANN, HF, TP, ULF, and LF/HF parameters was converted to a log_2_ that conformed to a normal distribution. The AUC for log_2_SDNN (Figure 2A), SDNN index (Figure 2B), RMSSD (Figure 2C), pNN50 (Figure 2D), and TR index (Figure 2E) was 0.811 (95% confidence interval [CI] 0.688–0.935), 0.820 (95% CI 0.671–0.968), 0.807 (95% CI 0.659–0.956), 0.813 (95% CI 0.673–0.952) and 0.807 (95% CI 0.659–0.956), respectively, in predicting the therapeutic response to metoprolol in POTS children. The cut-off values of the indices were 7.2 ms, 79.0 ms, 45.5 ms, 20.0 ms and 33.7 ms, respectively, yielding sensitivities of 73.5, 94.1, 61.8, 64.7, and 85.6%, respectively, and specificities of 100, 63.6, 90.9, 90.9, and 72.7%, respectively.

### Baseline HRV Frequency-Domain Indices Predict Short-Term Therapeutic Response to Oral Metoprolol in Children With POTS

**FIGURE 3:**
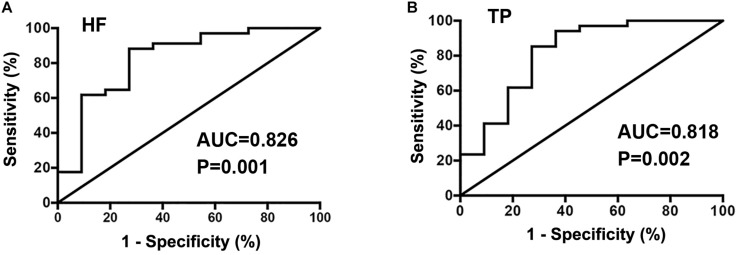
Receiver operator characteristic curve analysis of HRV frequency domain indexes of HF **(A)** and TP **(B)** as predictors of therapeutic response to metoprolol in children with POTS.

For the frequency-domain indices, the AUC values for HF (Figure 3A) and TP (Figure 3B) were 0.826 (95% CI 0.670–0.982) and 0.818 (95% CI 0.655–0.981), respectively, in predicting short-term therapeutic response to oral metoprolol. Cut-off values for the log_2_ indices were 10.1 and 11.8 ms^2^, respectively, yielding sensitivities of 88.2 and 85.3%, respectively, and specificities of 72.7 and 72.7%, respectively.

### Combined Baseline TR Index and SDNN Index for Predicting Outcome in Children With POTS

We showed that there was a strong correlation between HRV indicators. Please see the “Supplementary Table 1.” To find indicators with high sensitivity and specificity, we used series-parallel analysis of those indicators and found that the baseline TR index ≤ 33.7 and SDNN index ≤ 79.0 ms yielded sensitivity 85.3%, specificity 81.8% and accuracy 84.4% to predict response to metoprolol.

**FIGURE 4:**
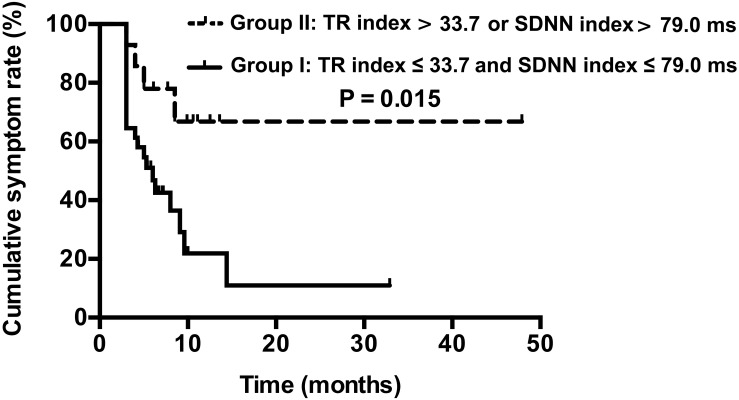
Kaplan–Meier curve analysis of cumulative symptom rate between two groups of children with POTS. Group I: TR index ≤ 33.7 and SDNN index ≤ 79.0 ms, *n* = 31; Group II: TR index > 33.7 and SDNN index ≤ 79.0 ms, TR index ≤ 33.7 and SDNN index > 79.0 ms, or TR index > 33.7 and SDNN index > 79.0 ms, *n* = 14.

In the second follow-up studies, based on the combined cut-off values for the TR index and SDNN index, the children with POTS were divided into those with TR index ≤ 33.7 and SDNN index ≤ 79.0 ms (group I; *n* = 31) and with TR index > 33.7 and SDNN index ≤ 79.0 ms, TR index ≤ 33.7 and SDNN index > 79.0 ms, or TR index > 33.7 and SDNN index > 79.0 ms (group II; *n* = 14). The Kaplan–Meier curves of the two groups were plotted. Cumulative symptom rates were 92.9, 78.6, 69.8, 69.8, and 69.8% at 3, 6, 9, 12 and 48 months, respectively, for children in group II and 64.5, 48.4, 39.6, 23.8, and 11.9% at 3, 6, 9, 12 and 48 months, respectively, for children in group I. Cumulative symptom rates were significantly higher for children with POTS in group II than group I (χ^2^ = 5.952, *P* = 0.015) (Figure 4).

## Discussion

In this study, we found that values for the baseline HRV frequency domain indicators ULF, VLF, LF, HF, and TP and time domain indicators were significantly lower for responders than non-responders to metoprolol for POTS; however LF/HF values were markedly greater than those of non-responders. On further ROC curve analysis of HRV index, the baseline SDNN, SDNN index, RMSSD, pNN50, TR index, HF and TP were of value in predicting the efficacy of metoprolol. To improve the sensitivity and specificity of the prediction of drug efficacy, we used series-parallel analysis of the indicators. The combined baseline TR index ≤ 33.7 and SDNN index ≤ 79.0 ms as cut-off values yielded sensitivity, specificity and accuracy of 85.3, 81.8 and 84.4%, respectively, to predict the therapeutic response to metoprolol. The cumulative symptom rate in patients was significantly higher for group II than group I patients (TR index ≤ 33.7 and SDNN index ≤ 79.0 ms) (*P* < 0.05). Therefore, combined baseline TR index and SDNN index could be used as preliminary measures to predict responsiveness to metoprolol for POTS in children.

Postural tachycardia syndrome belongs to chronic OI in children, and patients often report dizziness, headache, palpitations and fatigue, believed to result from transient decreased blood flow to the brain (Stewart et al., 2018; Spahic et al., 2019). Metoprolol, a β-blocker, is considered an effective method for treating childhood POTS (Bryarly et al., 2019). Responders in our study showed marked reduction in SS on metoprolol as compared with non-responders. However, we also found that when we did not choose to use β-blockers in POTS, the effectiveness was limited. Additionally, hypotension, fatigue, and reduced exercise capacity are potential side effects of β-blockers. Therefore, exploring a non-invasive indicator of HRV is extremely important to predict the efficacy of metoprolol in the treatment of POTS.

Previous studies have shown that time domain indices of HRV are useful measures of the changes in HR over time or the intervals between successive normal cardiac cycles, and they reflect alterations in autonomic tone that are predominantly vagally mediated (Young and Benton, 2018). The ULF and VLF component was proposed as an auxiliary marker for sympathetic modulation (Kainuma et al., 2014). The interpretation of the LF component is controversial. Some authors consider it a combination of sympathetic and parasympathetic modulation, but others as a measure of sympathetic modulations. Research also shows that β-blockade resulted in a reduced LF power. Therefore, in practical terms, an increased LF component has been generally considered to result from sympathetic modulation (Sztajzel, 2004). The HF component is generally defined as a marker of vagal modulation (Zaravko et al., 2015).

According to previous literature, there are two types of POTS: neuropathic and hyperadrenergic. Neuropathic POTS is mainly characterized by vasodilation with over-enhanced vagal tone; nevertheless, hyperadrenergic POTS is mainly characterized by increased plasma noradrenaline level caused by sympathetic over-enhancement. Therefore, as compared with healthy controls, our POTS children presented increased vagal and sympathetic nerve tone. Our study showed that as compared with normal controls, SDNN index, pNN50, T R index, LF and TP were significantly increased, which suggests that vagal and sympathetic nerve tone in POTS patients was increased to a certain extent, which is consistent with previous research.

The pathogenesis of POTS is still unclear, possibly including autonomic dysfunction (Shannon et al., 2000), excessive vasodilation (Liao et al., 2013), and low central blood volume (Zhang et al., 2012). Providing active and effective treatment to children with POTS is an important clinical issue to investigate. Previous studies suggested that metoprolol could achieve better therapeutic effects in children with high catecholamine level, high adrenergic status, and β-receptor hypersensitivity (Thieben et al., 2007). To predict responders to metoprolol before treatment, we used to test plasma norepinephrine, copeptin and CNP levels, attempting to find predictive measures for therapeutic responders to metoprolol before treatment of children with POTS (Benditt and Chen, 2012; Zhang et al., 2014; Zhao et al., 2014; Lin et al., 2015b). However, the instability of plasma norepinephrine, the complexity of plasma copeptin detection and the invasiveness of CNP detection limited their clinical use to a certain extent.

Heart rate variability as a predictive measure reflecting the modulation of autonomic nervous function with the advantages of non-invasiveness and easy to perform was hypothesized to help in predicting the therapeutic response to metoprolol in treating POTS in children (Zygmunt and Stanczyk, 2004). The immediate HR is the result of the interaction between the sympathetic and vagal nerves, which suggests that the two are constantly coordinated to produce a difference in HR. HRV reflects changes in HR over a long period. When the HRV is within a certain range, the vagal-sympathetic nerve system is well-regulated. That is to say, the child has a certain reserve capacity in the nervous system. Nevertheless, when it functions beyond the reserve capacity, it will manifest as a clinical symptom dominated by sympathetic or vagal nerves. In the present study, we employed 24-h HRV analysis to see if it is useful for predicting the therapeutic response to metoprolol in pediatric POTS. The reason that we used 24-h HRV analysis, including daytime HRV data, is that for POTS cases, the pathogenesis is closely related to postural change from supine to upright and such a postural change often occurs during the daytime. Actually, patients with POTS have the disproportionately enhanced sympathetic activity and vagal withdrawal during sitting or standing (Baker et al., 2018), which are often at daytime. Such alterations in autonomic nervous system function in children with POTS were also demonstrated during the HUTT (Stewart, 2000). While, we did not find any differences in nighttime HRV between POTS and normal controls, which accorded with the findings in previous studies (Mallien et al., 2014), nor between responders and non-responders to metoprolol. The above facts support us to use 24-h HRV recording to analyze the abnormal autonomic nervous function in children with POTS to predict therapeutic response to metoprolol in pediatric POTS. Of course, we should pay attention to the fact that 24-h HRV is also influenced by physical activities on daytime in the clinical evaluation.

To examine whether baseline 24-h HRV indices could predict the therapeutic response to metoprolol in children with POTS before treatment, we found that LF/HF ratio were higher in responders but other HRV indicators were lower than in non-responders before metoprolol treatment. The results suggested that the baseline indicators of sympathetic and vagal nervous tone were significantly lower in responders than non-responders before metoprolol treatment, but the ratio of sympathetic to vagal nervous modulation was significantly higher in responders than non-responders before metoprolol treatment. That is to say, before metoprolol treatment for POTS, responders to metoprolol had a predominant baseline sympathetic nervous modulation as compared with non-responders. Also, metoprolol achieves its therapeutic effects by inhibiting excessive sympathetic modulation by blocking beta receptors (Mizumaki, 2011).

Additionally, we further showed that when we combined TR index ≤ 33.7 and SDNN index ≤ 79.0 ms as the cut-off values to predict the short-term efficacy of metoprolol in children with POTS, the sensitivity, specificity, and accuracy were 85.3, 81.8, and 84.4%, respectively. We further confirmed that the combined TR index ≤ 33.7 and SDNN index ≤ 79.0 ms were useful as effective predictors for long-term outcome after metoprolol treatment. Therefore, HRV indicators may be non-invasive and easy-to-use predictors that could be used to predict the efficacy of metoprolol for POTS in children before the implication of the treatment.

However, our research still had limitations. This study was from a single center, and the case number was not sufficiently large. Also, sympathetic nervous activity was not directly measured. The 24-h HRV is influenced by the physical activities on daytime, which needs the patients to avoid strenuous exercise and emotional excitement. The present study provided non-invasive and easy-to-use effective predictors for the therapeutic response to metoprolol in children with POTS, which would provide great help for deciding whether to choose metoprolol for pediatric POTS. In the future, multi-center studies should be conducted to validate the study findings.

## Data Availability Statement

The datasets generated for this study are available on request to the corresponding author.

## Ethics Statement

The studies involving human participants were reviewed and approved by the Ethics Committee of the First Hospital of Peking University (2018 [202]). Written informed consent to participate in this study was provided by the participants legal guardian/next of kin.

## Author Contributions

JD, HJ, CT, and YYW conceived and designed the study. YYW, CZ, SC, and PL acquired the data. YYW and CZ analyzed and interpreted the data. YYW, HJ, and JD drafted the manuscript. CZ, SC, PL, and YLW revised the manuscript for important intellectual content. YYW, CZ, SC, PL, YLW, CT, JD, and HJ approved the final version to be submitted.

## Conflict of Interest

The authors declare that the research was conducted in the absence of any commercial or financial relationships that could be construed as a potential conflict of interest.
