# Supplementary material for: Heart Rate Variability Predicts Therapeutic Response to Metoprolol in Children With Postural Tachycardia Syndrome
Source: Front Neurosci. 2019 Nov 12;13:1214. doi: 10.3389/fnins.2019.01214 (PMC6861190; doi:10.3389/fnins.2019.01214)
Supplement: Supplementary file 1 [file Table_1.docx]

**Supplemental table 1. Correlation analysis between HRV indicators in children with POTS**

| HRV indices | | SDANN ^a^, ms | SDNN index, ms | RMSSD^a^, ms | pNN50, % | TR index | ULF ^a^, ms^2^ | VLF, ms^2^ | LF ^a^, ms^2^ | HF ^a^, ms^2^ | TP ^a^, ms^2^ | LF/HF ^a^ |
| --- | --- | --- | --- | --- | --- | --- | --- | --- | --- | --- | --- | --- |
| SDNN, ms | r | 0.893 | 0.785 | 0.786 | 0.725 | 0.707 | 0.932 | 0.789 | 0.681 | 0.729 | 0.773 | -0.542 |
|  | P | 0.000 | 0.000 | 0.000 | 0.000 | 0.000 | 0.000 | 0.000 | 0.000 | 0.000 | 0.000 | 0.000 |
| SDANN ^a^, ms | r | 1 | 0.553 | 0.658 | 0.496 | 0.491 | 0.859 | 0.607 | 0.557 | 0.615 | 0.658 | -0.456 |
|  | P | - | 0.000 | 0.000 | 0.001 | 0.001 | 0.000 | 0.000 | 0.000 | 0.000 | 0.000 | 0.002 |
| SDNN index, ms | r | 0.553 | 1 | 0.939 | 0.944 | 0.736 | 0.747 | 0.923 | 0.874 | 0.904 | 0.934 | -0.662 |
|  | P | 0.000 | - | 0.000 | 0.000 | 0.000 | 0.000 | 0.000 | 0.000 | 0.000 | 0.000 | 0.000 |
| RMSSD ^a^, ms | r | 0.658 | 0.939 | 1 | 0.957 | 0.9644 | 0.695 | 0.801 | 0.843 | 0.963 | 0.918 | -0.813 |
|  | P | 0.000 | 0.000 | - | 0.000 | 0.000 | 0.000 | 0.000 | 0.000 | 0.000 | 0.000 | 0.000 |
| pNN50, % | r | 0.496 | 0.944 | 0.957 | 1 | 0.733 | 0.665 | 0.803 | 0.801 | 0.925 | 0.891 | -0.778 |
|  | P | 0.000 | 0.000 | 0.000 | - | 0.000 | 0.000 | 0.000 | 0.000 | 0.000 | 0.000 | 0.000 |
| TR index | r | 0.491 | 0.736 | 0.9644 | 0.733 | 1 | 0.635 | 0.628 | 0.527 | 0.658 | 0.638 | -0.573 |
|  | P | 0.001 | 0.000 | 0.000 | 0.000 | - | 0.000 | 0.000 | 0.000 | 0.000 | 0.000 | 0.000 |
| ULF ^a^, ms^2^ | r | 0.859 | 0.747 | 0.695 | 0.665 | 0.635 | 1 | 0.754 | 0.589 | 0.661 | 0.701 | -0.515 |
|  | P | 0.000 | 0.000 | 0.000 | 0.000 | 0.000 | - | 0.000 | 0.000 | 0.000 | 0.000 | 0.000 |
| VLF, ms^2^ | r | 0.607 | 0.923 | 0.801 | 0.803 | 0.628 | 0.754 | 1 | 0.803 | 0.780 | 0.876 | -0.522 |
|  | P | 0.000 | 0.000 | 0.000 | 0.000 | 0.000 | 0.000 | - | 0.000 | 0.000 | 0.000 | 0.000 |
| LF ^a^, ms^2^ | r | 0.557 | 0.874 | 0.843 | 0.801 | 0.527 | 0.589 | 0.803 | 1 | 0.885 | 0.946 | -0.552 |
|  | P | 0.000 | 0.000 | 0.000 | 0.000 | 0.000 | 0.000 | 0.000 | - | 0.000 | 0.000 | 0.000 |
| HF ^a^, ms^2^ | r | 0.615 | 0.904 | 0.963 | 0.925 | 0.658 | 0.661 | 0.78 | 0.885 | 1 | 0.956 | -0.853 |
|  | P | 0.000 | 0.000 | 0.000 | 0.000 | 0.000 | 0.000 | 0.000 | 0.000 | - | 0.000 | 0.000 |
| TP ^a^, ms^2^ | r | 0.658 | 0.934 | 0.918 | 0.891 | 0.638 | 0.701 | 0.876 | 0.946 | 0.956 | 1 | -0.701 |
|  | P | 0.000 | 0.000 | 0.000 | 0.000 | 0.000 | 0.000 | 0.000 | 0.000 | 0.000 | - | 0.000 |

*HRV, Heart rate variability; POTS,* *postural tachycardia syndrome; SDANN, standard deviation of the average of NN intervals in all 5-min segments of the entire recording; SDNN index, mean of the standard deviation of NN intervals for each 5-min segment; RMSSD, root mean square of the successive NN interval difference; pNN50, percentage of adjacent RR intervals > 50 ms; TR index, triangular index; ULF, ultra low frequency; VLF, very low frequency; LF, low frequency; HF, high frequency; TP, total power; LF/HF, ratio of low to high frequency power. ^a^ Non-normal distribution.*
